# CausalCervixNet: convolutional neural networks with causal insight (CICNN) in cervical cancer cell classification—leveraging deep learning models for enhanced diagnostic accuracy

**DOI:** 10.1186/s12885-025-13926-2

**Published:** 2025-04-03

**Authors:** Zahra Taghados, Zohreh Azimifar, Malihezaman Monsefi, Mojgan Akbarzadeh Jahromi

**Affiliations:** 1 Department of Computer Science, Engineering and Information Technology, Shiraz University, Shiraz, Iran; 2 Department of Biology, Shiraz University, Shiraz, Iran; 3 Department of Pathology, Shiraz University of Medical Sciences, Shiraz, Iran

**Keywords:** Causal discovery, Causal inference, Causal reasoning, Causality, Cervical cancer, Classification, Deep learning, Machine learning

## Abstract

Cervical cancer is a significant global health issue affecting women worldwide, necessitating prompt detection and effective management. According to the World Health Organization (WHO), approximately 660,000 new cases of cervical cancer and 350,000 deaths were reported globally in 2022, with the majority occurring in low- and middle-income countries. These figures emphasize the critical need for effective prevention, early detection, and diagnostic strategies. Recent advancements in machine learning (ML) and deep learning (DL) have greatly enhanced the accuracy of cervical cancer cell classification and diagnosis in manual screening. However, traditional predictive approaches often lack interpretability, which is critical for building explainable AI systems in medicine. Integrating causal reasoning, causal inference, and causal discovery into diagnostic frameworks addresses these challenges by uncovering latent causal relationships rather than relying solely on observational correlations. This ensures greater consistency, comprehensibility, and transparency in medical decision-making.

This study introduces CausalCervixNet, a Convolutional Neural Network with Causal Insight (CICNN) tailored for cervical cancer cell classification. By leveraging causality-based methodologies, CausalCervixNet uncovers hidden causal factors in cervical cell images, enhancing both diagnostic accuracy and efficiency. The approach was validated on three datasets: SIPaKMeD, Herlev, and our self-collected ShUCSEIT (Shiraz University-Computer Science, Engineering, and Information Technology) dataset, containing detailed cervical cell cytopathology images. The proposed framework achieved classification accuracies of 99.14%, 97.31%, and 99.09% on the SIPaKMeD, Herlev, and ShUCSEIT datasets, respectively.

These results highlight the importance of integrating causal discovery, causal reasoning, and causal inference into diagnostic workflows. By merging causal perspectives with advanced DL models, this research offers an interpretable, reliable, and efficient framework for cervical cancer diagnosis, contributing to improved patient outcomes and advancements in cervical cancer treatment.

## Introduction

Cervical cancer remains a leading cause of morbidity and mortality among women worldwide, ranking as the fourth most prevalent cancer among females [1]. Human papillomavirus (HPV) infection accounts for approximately 90% of cases [2], with 604,000 new diagnoses and 342,000 deaths reported globally in 2020 alone [3]. Effective screening programs, including routine Pap smears and HPV vaccinations, are crucial in reducing disease burden and improving survival rates [4, 5].

Advancements in computational methodologies have significantly enhanced cervical cancer screening, particularly through automated classification of cytological images [6]. Traditional machine learning (ML) approaches have demonstrated considerable efficacy in recognizing cellular abnormalities; however, they predominantly rely on correlation-based feature learning, which assumes that training and test data share similar statistical distributions [7, 8]. This assumption often fails in real-world clinical settings where data variability and patient heterogeneity challenge model generalizability.

The limitations of correlation-driven ML models, particularly their lack of interpretability and susceptibility to bias, pose significant barriers to clinical adoption [9]. While these models excel at pattern recognition, they do not inherently capture causal relationships between pathological features and clinical outcomes. As Pearl and Mackenzie [10] emphasized, 'correlation does not imply causation,' underscoring the necessity of transitioning toward causality-based methodologies. Medical decision-making demands more than associative evidence; it requires an understanding of underlying causal mechanisms to improve reliability and trust in AI-driven diagnostics [11, 12].

Causal discovery has emerged as a pivotal area of research, building on foundational work by Fisher (1970) and Granger (1969) and further refined by Pearl (2011), who formalized a structured framework for causal inference. These advancements have facilitated the development of methods capable of extracting cause-and-effect relationships from observational data, particularly in contexts where controlled experimentation is impractical or ethically constrained [13, 14]. By integrating causal inference techniques into AI-driven medical imaging, researchers aim to improve model robustness, enhance diagnostic accuracy, and provide interpretable insights critical for clinical decision-making.

This study introduces CausalCervixNet, an advanced deep learning framework that incorporates causal inference techniques for more accurate classification of cervical cytology images. By leveraging a structured approach to causal reasoning, CausalCervixNet transcends the limitations of conventional ML models, offering enhanced interpretability and diagnostic precision. To validate its efficacy, the model was evaluated on three diverse datasets—SIPaKMeD, Herlev, and ShUCSEIT—each containing high-resolution cervical cytopathology images representative of varied clinical conditions.

A critical component of this approach is training deep learning models on both normal and malignant cell images to facilitate accurate differentiation. This ensures that the model captures salient morphological features, mitigates bias, and generalizes effectively to unseen clinical data, ultimately enhancing its applicability in real-world diagnostic settings.

The key contributions of this study are:

1. Advancing causality-driven AI in medical imaging by introducing a novel framework tailored for cervical cytology classification.
2. Overcoming the limitations of conventional ML models by integrating causal inference methodologies that enhance interpretability and robustness.
3. Developing a deep learning model that identifies and leverages causal factors influencing classification outcomes, thereby improving diagnostic reliability.
4. Providing a high-resolution, multi-source cervical cytopathology dataset, expanding opportunities for future research in AI-driven cancer diagnostics.
5. Demonstrating superior classification performance, particularly on the SIPaKMeD dataset, highlighting the efficacy of causal modeling in improving diagnostic accuracy.

The structure of this paper is as follows: the Literature Review examines conventional classification techniques and recent advancements in causal inference. The Background contextualizes key theoretical principles. The Methodology outlines the experimental design and implementation details. The Results section presents quantitative and qualitative performance evaluations. The Discussion provides a critical analysis of findings, implications, and potential areas for further research. Finally, the Conclusion synthesizes key insights and outlines future directions for integrating causality-driven AI methodologies in clinical practice.

By pioneering causality-based AI in cervical cytology, this study aims to bridge the gap between computational innovation and clinical applicability, fostering more accurate, interpretable, and trustworthy diagnostic solutions.

## Literature review

In this section, we provide a brief review and discussion of the previous related works, which can be categorized into cervical cells classification, causal discovery, and causal inference.

Cervical cell classification plays a crucial role in computer-aided cervical cancer detection, with recent advancements in the field leading to significant progress. Chen et al. [15] emphasize its importance, while [16] address the challenge of limited data availability by fine-tuning pre-trained deep learning models from ImageNet datasets and employing the deep feature fusion (DFF) technique for improved classification performance. DeepPap, introduced by [17], automatically extracts hierarchical features from cellular image patches, eliminating the need for manual cytoplasm/nucleus segmentation and hand-crafted features, resulting in more accurate results. Fang et al. [18] propose DeepCELL, a deep convolutional neural network that captures feature information from cervical cytology images using multiple kernels with different sizes, enhancing accuracy and timeliness in cervical cancer diagnosis. Liu et al. [19] introduce CVM-Cervix, a novel framework combining CNN, Visual Transformer, and Multilayer Perceptron. Notably, it tackles an 11-class classification task, the most complex in the existing literature, and significantly improves overall classification performance. Zhao et al. [20] highlight the significance of cervical cell classification in early-stage cervical cancer screening. Their proposed model combines taming transformers with T2T-ViT to address imbalanced datasets and uneven image quality challenges, incorporating techniques like upweighting classes with few samples and generating samples using CCG-taming transformers, yielding insightful results and recommendations for enhanced cervical cancer classification. Deo et al. [21] present CerviFormer, an innovative approach leveraging cross attention and latent transformer techniques for classification, surpassing other alternative classifier models in terms of accuracy, precision, and recall for the 3/2 target classes. Fekri-Ershad et al. [22] propose a novel machine learning strategy involving a tuned three-layer perceptron fed with trained deep convolutional neural networks for feature extraction and classification. Fang et al. [23] propose a deep integrated fusion method that combines local and global features for cervical cell classification. Their method enhances the robustness of feature extraction by leveraging a multi-scale approach, which improves classification accuracy in challenging datasets. Interpretable Cervical Cell Classification: A Comparative Analysis [24] conduct a comparative analysis of interpretable cervical cell classification models, emphasizing the trade-offs between accuracy and model explainability, particularly in medical diagnostic contexts where transparency is critical. Anand et al. [25] introduce CervicalNet, a convolutional neural network optimized for five-class cervical cell classification. By integrating attention mechanisms, CervicalNet improves feature relevance and significantly enhances classification performance.

Recent literature in cervical cell classification uses deep learning models, novel architectures, and advanced techniques to improve accuracy in computer-aided cervical cancer detection, offering promise for early detection and treatment.

Causal discovery identifies causal relationships between variables based on observational data [14]. Zhang et al. [26] present SCIT, a fast method for testing conditional independence in linear structural equation models using kernel functions and permutation tests. They also discuss the HSIC formula for measuring independence in kernel-based methods. Zhang et al. [27] propose two kernel-based methods, HSIC and KCI tests, for testing independence and conditional independence between continuous variables, showcasing their efficiency for high-dimensional data. Rast [28] introduce an algorithm to uncover latent graph structures in biological systems based on experimental data, holding potential for applications in medicine and biotechnology. Zheng et al. [29] present Causal-learn, an open-source Python library for causal discovery and inference. It includes scalable algorithms for learning causal structures, testing independence, and evaluating causal effects, making it a versatile tool for both researchers and practitioners. Wan et al. [30] provide a comprehensive survey exploring the integration of causal discovery with large language models. This study discusses how language models can leverage causal inference to enhance interpretability and improve robustness, particularly in applications that process complex linguistic or textual data.

Causal inference has been a pivotal research topic across domains for several decades [31, 32]. In the context of [33], statistical causal inference (SCI) methods aim to estimate causal effects from observational data when randomized controlled trials are not possible, making it crucial for public health interventions. Glass et al. [34] emphasize the significance of identifying causal associations in public health and offer guidelines for interpreting evidence. They highlight the importance of employing rigorous study designs and statistical methods to establish causality. Furthermore, the authors present examples of successful interventions that were built upon causal associations in the field of public health. In "Practical Causal Inference for Ecoepidemiologists" by Fox [35], a systematic approach is presented to assess the link between environmental factors and observed effects. The work highlights the importance of recognizing uncertainty and limitations in scientific knowledge for informed environmental management decisions. Liu et al. [36] introduce CIIC, a novel framework for image captioning that integrates causal intervention into both object detection and caption generation processes, effectively mitigating the confounding effect. Furthermore, Lopez-Paz et al. [37] aim to find causal signals in images by identifying observable footprints that reveal causal relationships between object categories in static image collections through a learning approach. Terziyan et al. [38] introduce CA-CNN, a CNN architecture with a causality map capturing relationships between features in images and other data channels. CA-CNN autonomously identifies crucial causalities for accurate classification, enhancing accuracy in datasets where class distribution depends on causal scene characteristics. Causal inference techniques have also benefited from recent computational advancements. Gao et al. [39] provide a survey on causal inference in recommender systems, highlighting its potential to address biases, confounding factors, and fairness in recommendations. The study emphasizes how causal approaches can improve the reliability and fairness of personalized systems. Similarly, Luo et al. [40] offer a detailed review of causal inference techniques in recommendation systems, presenting methodologies to estimate user preferences while mitigating spurious correlations.

This literature review demonstrates the diverse applications of causal inference and its significance in various domains, emphasizing the importance of rigorous methodologies to establish causality and make informed decisions in different fields.

### Advantages and disadvantages of existing literature

The existing literature on cervical cell classification and causality demonstrates significant advancements but also reveals notable limitations. Recent studies effectively integrate advanced deep learning models, such as CNNs, transformers, and hybrid frameworks, leading to improved classification performance and robustness [17–19]. Methods like DeepPap, DeepCELL, and CervicalNet showcase the benefits of deep feature extraction and attention mechanisms, achieving high accuracy even with imbalanced datasets [17, 18, 25]. Additionally, the integration of causal reasoning and causal inference into classification workflows has enhanced model interpretability, providing insights into feature dependencies and addressing critical challenges in explainable AI [29, 37, 38]. Techniques like SCIT, HSIC, and CIIC have extended causal reasoning to high-dimensional data, while the use of benchmark datasets such as SIPaKMeD and Herlev has standardized the evaluation of these models [26–28]. However, these advancements are accompanied by several limitations. The datasets often lack real-world complexity, such as overlapping cells and variable staining, reducing the generalizability of trained models [15, 16]. While causal methodologies enhance transparency, their integration with deep learning frameworks increases computational complexity and may limit scalability for large-scale applications [29, 30, 39]. Furthermore, the focus on curated datasets and controlled environments restricts real-world validation and clinical integration [20, 23]. The absence of standardized evaluation metrics for models combining causality and classification also complicates comparative analysis [14, 24, 40]. Despite these challenges, the integration of causal reasoning with advanced deep learning techniques presents an opportunity to address these gaps, particularly through unified frameworks validated on diverse datasets.

## Background

### Causality

Causality is the fundamental principle that links events together. It represents the recognition that one event, known as the cause, gives rise to another event, referred to as the effect [41]. This cause-and-effect relationship is crucial in various fields, helping us comprehend how things work and make informed decisions based on these connections [42]. However, establishing causality can be complex due to multiple factors, like confounding variables and intricate interactions among variables. Untangling causality demands a rigorous approach [43]. Despite these challenges, grasping causality offers valuable insights, fostering a deeper understanding of the world and empowering us to predict future outcomes based on past observations [44].

The Hilbert–Schmidt Independence Criterion (HSIC) and Kernel Conditional Independence (KCI) are pivotal concepts in causality and statistical independence within machine learning and causal inference. These notions serve as essential tools in modern machine learning and causal inference, enabling researchers and practitioners to identify causal relationships, handle non-linear dependencies among variables, and enhance the practical application of causality across domains. Leveraging HSIC and KCI equips contemporary machine learning approaches to manage intricate variable relationships, resulting in significant strides in comprehending causal structures and their effects on real-world situations [27].

#### Hilbert–Schmidt Independence Criterion

The Hilbert–Schmidt Independence Criterion (HSIC) is a statistical measure widely used to assess the dependence between two random variables or datasets [45]. It is calculated as the squared inner product between the cross-covariance operator of X and Y within their respective Reproducing Kernel Hilbert Spaces (RKHSs). The formula for HSIC is:

1 $$HSIC(X,Y) = \frac{1}{n-1} tr(K\_H L\_H K\_L L\_L)$$

Here, n represents the sample size, K_H and K_L are the kernel matrices for X and Y, and L_H and L_L are the centering matrices. The 'tr' represents the trace operator. Kernel matrices are constructed by evaluating a kernel function on pairs of data points from X and Y, transforming the data into a high-dimensional feature space where the inner product signifies their similarity. The centering matrices, L_H and L_L, are essential in HSIC computation as they ensure a mean of zero for each kernel matrix [46]. HSIC compares the joint distribution of the features of X and Y with the product of their marginal distributions. A HSIC value of zero indicates independence between X and Y, while a positive value suggests a non-zero and higher dependence [47].

#### Kernel Conditional Independence

The Kernel Conditional Independence Test (KCIT) is a valuable approach for evaluating the conditional independence of continuous variables. It utilizes a test statistic derived from the uncorrelatedness of functions within suitable RKHSs [26]. Consider three random variables, X, Y and Z, with a joint distribution P (X, Y, Z). The goal is to test whether X and Y are conditionally independent given Z, meaning that P (X, Y | Z) = P (X | Z) P (Y | Z). The KCIT compares the empirical conditional distribution of X given Z and Y given Z to the product of their marginal distributions. This is achieved by using kernel density estimators to estimate these distributions and comparing them using a test statistic.

The KCIT statistic is defined as:

2 $$KCI (X, Y|Z) = \frac{1}{{n}^{2}} tr (KH\_X KH\_Y KH\_Z)$$

Here, n represents the sample size, tr denotes the trace of a matrix, and KH_X, KH_Y, and KH_Z are the centered kernel matrices associated with X, Y and Z, respectively [27, 48]. A value of zero indicates conditional independence, while a larger value suggests a stronger dependence. KCIT is widely employed in causal discovery and conditional independence testing to assess the relationships between variables in the presence of a conditioning variable.

### Causality map

The causality map is a visual representation of estimated pairwise causal relationships between features extracted from image [38]. Following the fundamental principle of conditional probability, it is linked with the concept of joint probability:

3 $$P\left(F^i\vert F^j\right)=\frac{(\max_{a,b=1,k}F_{a,b}^i)(\max_{a,b=1,k}F_{a,b}^j)}{\sum_{a,b=1}^kF_{a,b}^j}$$

The feature maps exclusively consist of non-negative numbers, owing to the use of ReLU operations, serving as indicators of the presence of a specific feature within a given batch (location in the image). By normalizing the values of the feature maps to the [0, 1] interval through division by the maximum possible value of feature presence, we can interpret these values as probabilities. As illustrated in Fig. 2, the features${F}^{1}$,${F}^{2}$,…,${F}^{n}$ represented by k × k feature maps, are leveraged to compute each$P\left({F}^{i}|{F}^{j}\right)$. Equation 3 yields a value within the interval [0, 1], providing a robust estimate for conditional probability. It considers the joint probability, which signifies the highest presence of both features within the image (each in their respective location). The variables a and b serve as indices denoting positions within a k-dimensional value matrix.

The causality map serves as a pivotal advancement, enhancing classification accuracy for image datasets. It proves particularly valuable for datasets where the distribution of images across classes hinges on the causal relationships inherent in the scenes depicted.

### Transfer learning

The utilization of Convolutional Neural Networks (CNNs) in AI for medical diagnosis, particularly in medical image classification, has had a profound impact [49]. Advancements in artificial intelligence, especially in deep learning techniques, have significantly contributed to the identification, classification, and quantification of patterns in medical images, making deep learning one of the most rapidly evolving domains within AI, with widespread and effective applications across various sectors. CNNs have emerged as the most prevalent and noteworthy deep learning architecture, representing a critical breakthrough in enabling autonomous detection of essential features without human intervention. Research consistently demonstrates that CNNs exhibit robustness to image noise and invariance to translation, rotation, and size, enhancing their object analysis capabilities [16, 49, 50].

Transfer learning (TL) using convolutional neural networks enhances performance on novel tasks by leveraging knowledge acquired from similar tasks learned beforehand. This approach is a significant breakthrough in medical image analysis, addressing challenges posed by data scarcity and optimizing time and hardware resources [51]. TL models are trained on large datasets like ImageNet [52], and their parameters can be used in custom neural networks for other related applications. TL techniques offer a solution to handle unseen data and limited data in clinical practice, as traditional neural networks may struggle with such data. Pre-trained networks, widely used for image classification in medical domains, reduce training time and minimize generalization errors due to their extensive training on ImageNet dataset comprising 1000 object categories [53].

For the cervical cell dataset, four pre-trained models—XceptionNet, VGG16, VGG19, and ResNet50—were employed. These models have already learned generic features from various datasets. Fine-tuning these models on the cervical cell dataset allows them to learn specific features relevant to this medical domain, leading to improved generalization and reduced training time and errors [16, 54].

- VGGNet represents a convolutional neural network architecture distinguished by its remarkable depth, and notable implementation of compact 3 × 3 convolution filters across the network. Such architectural attributes have substantially contributed to its outstanding performance, propelling VGGNet to the forefront of the ImageNet Challenge 2014, where it attained state-of-the-art results [55].
- ResNet50 is a convolutional neural network variant utilized in deep learning for image classification. It consists of 50 layers and has been extensively trained on a large image dataset to recognize patterns and features. ResNet50 effectively addresses the vanishing gradient problem in deep neural networks by employing residual connections. These connections enable the network to learn residual functions, simplifying the acquisition of complex image features and patterns [56].
- XceptionNet is a powerful deep learning architecture that efficiently combines depthwise separable convolutions and convolutional neural networks. It has 36 convolutional layers forming the feature extraction base, organized into 14 modules with linear residual connections. Its linear stack enables easy definition and modification [57].

## Method

### Classification with deep features

**Fig. 1:**
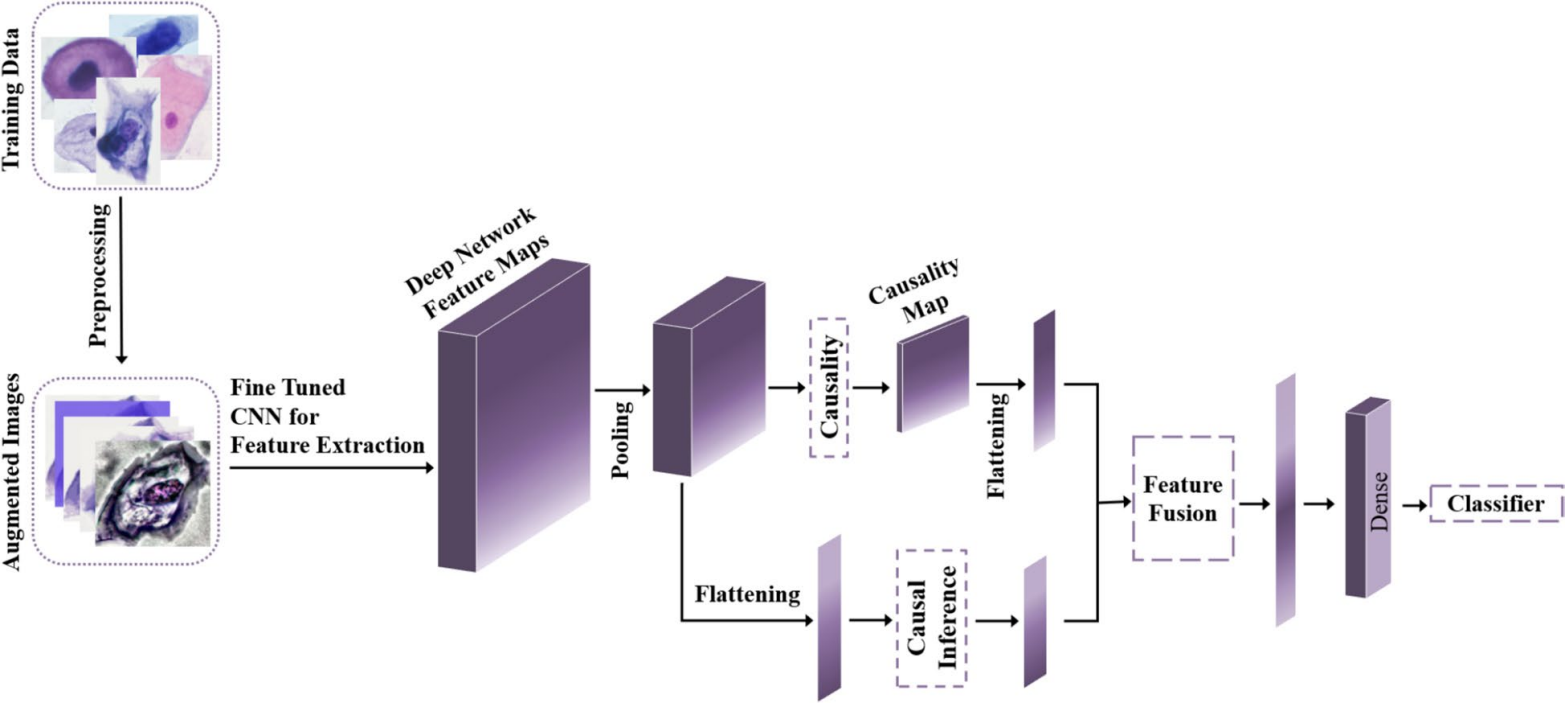
The CausalCervixNet framework begins with a preprocessing step where augmented images are generated using geometric transformations, color space transformations, kernel filters, random erasing, and image mixing. After preprocessing, the augmented images are inputted into a deep learning model, to extract feature maps. Following the final pooling layer, the network progresses through two key phases: 1) constructing a causality map containing estimations of pairwise causal relationships between features, and 2) flattening the feature maps while identifying causal factors associated with the target variable (y) using a novel causal inference scheme. The model's performance is evaluated using unseen test images, and assessed in terms of precision, recall, F1 score, and accuracy

In this study, we propose a novel classification approach leveraging Convolutional Neural Networks with Causal Insight (CICNN) to enhance the diagnostic accuracy of cervical cancer cell classification. The overall workflow of the CICNN model is illustrated in Fig. 1, which outlines the key stages of the methodology: preprocessing, feature extraction, causality estimation, causal inference, and classification.

Workflow Overview (Fig. 1): The CICNN model begins with preprocessing the dataset of microscopic images to prepare it for training. The preprocessed images are passed through a fine-tuned CNN to extract high-dimensional feature maps, which serve as the foundation for the subsequent causality analysis. Following the last pooling layer, the extracted feature maps are processed in two parallel directions:

1. Causality Estimation: A causality map is constructed by computing pairwise conditional probabilities between features, as detailed in Eq. (3).
2. Causal Inference: Flattened feature maps are analyzed to identify causal factors influencing the target label through independence and conditional independence testing (Eqs. 1 and 2).

The causal factors identified are fused with the original feature maps and fed into dense layers for final classification. This integration of causal insights significantly enhances the model’s interpretability and accuracy, especially for medical image datasets where relationships among features are often governed by causal dependencies. The CICNN methodology is summarized in the following pseudo-code, which outlines the main steps of the approach:

- Input: Raw microscopic images X = {${\text{x}}_{1}$,${\text{x}}_{2}$,…, ${\text{x}}_{\text{N}}$}, Labels Y = {${\text{y}}_{1}$, ${\text{y}}_{2}$,…, ${\text{y}}_{\text{N}}$}, where N = Number of cells.
- Preprocessing:
  - ◦ Segment images to isolate individual cells.
  - ◦ Apply augmentation techniques (e.g., rotation, flipping, normalization).
  - ◦ Prepare the data for CNN input.
- Feature Extraction: Use a fine-tuned CNN to compute feature maps F = {${F}^{1}$, ${F}^{2}$,…, ${F}^{N}$} for all input images.
- Causality Estimation:
  - ◦ For each pair of feature maps (${F}^{\text{i}}$, ${F}^{\text{j}}$), calculate the conditional probability P (${F}^{\text{i}}$∣${F}^{\text{j}}$) using Eq. (3).
  - ◦ Construct a causality map representing pairwise causal relationships.
- Causal Inference:
  - ◦ Perform independence testing for each feature map ${F}^{\text{i}}$ using Eq. (1).
  - ◦ Eliminate features independent of the target label Y.
  - ◦ Apply conditional independence testing (Eq. 2) to identify causal relationships between features and the target label.
- Feature Fusion: Combine causal factors with feature maps at the concatenation layer.
- Classification: Pass the fused features through dense layers to classify each image into one of the categories.
- Output: Classification results for all images.

**Fig. 2:**
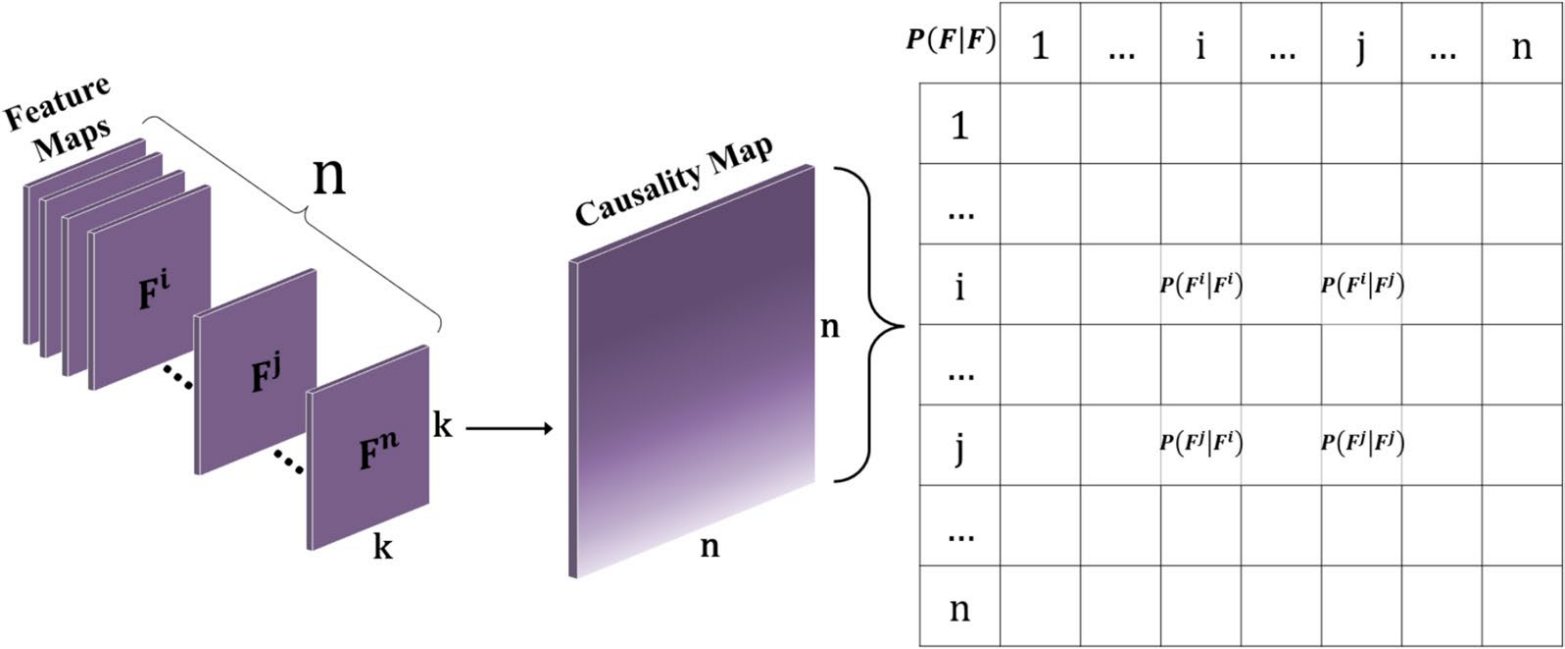
By utilizing feature maps from the ultimate pooling layer, a causality map is constructed, offering estimates for the pairwise causal relationships between features via the application of joint probability

In this study, our approach involves the extraction of feature maps from deep networks. Following the last pooling layer, we proceed in two directions. Firstly, we utilize these feature maps to construct a causality map, which learns pairwise conditional probabilities, a process commonly known as causality estimation, for features. Secondly, we aim to unveil the causal factors that impact the target label. The construction of the causality map follows the method outlined in the background (Eq. 3, Fig. 2). In the phase dedicated to identifying causal factors affecting the target variable, denoted as label, we implement a causal inference scheme by flattening feature maps. A causal factor, in this context, can signify a cause, effect, or independence with respect to y. Our process commences with an independence test between y and each factor to eliminate those demonstrating independence from y. In causal discovery, evaluating the dependencies of variables helps to identify causal links between variables. Employing the HSIC core independence test (Eq. 1), we infer statistical dependencies from the samples. After the removal of independent factors from y, we proceed with a conditional independence test to uncover the causal relationship between y and the remaining factors (Eq. 2).

**Fig. 3:**
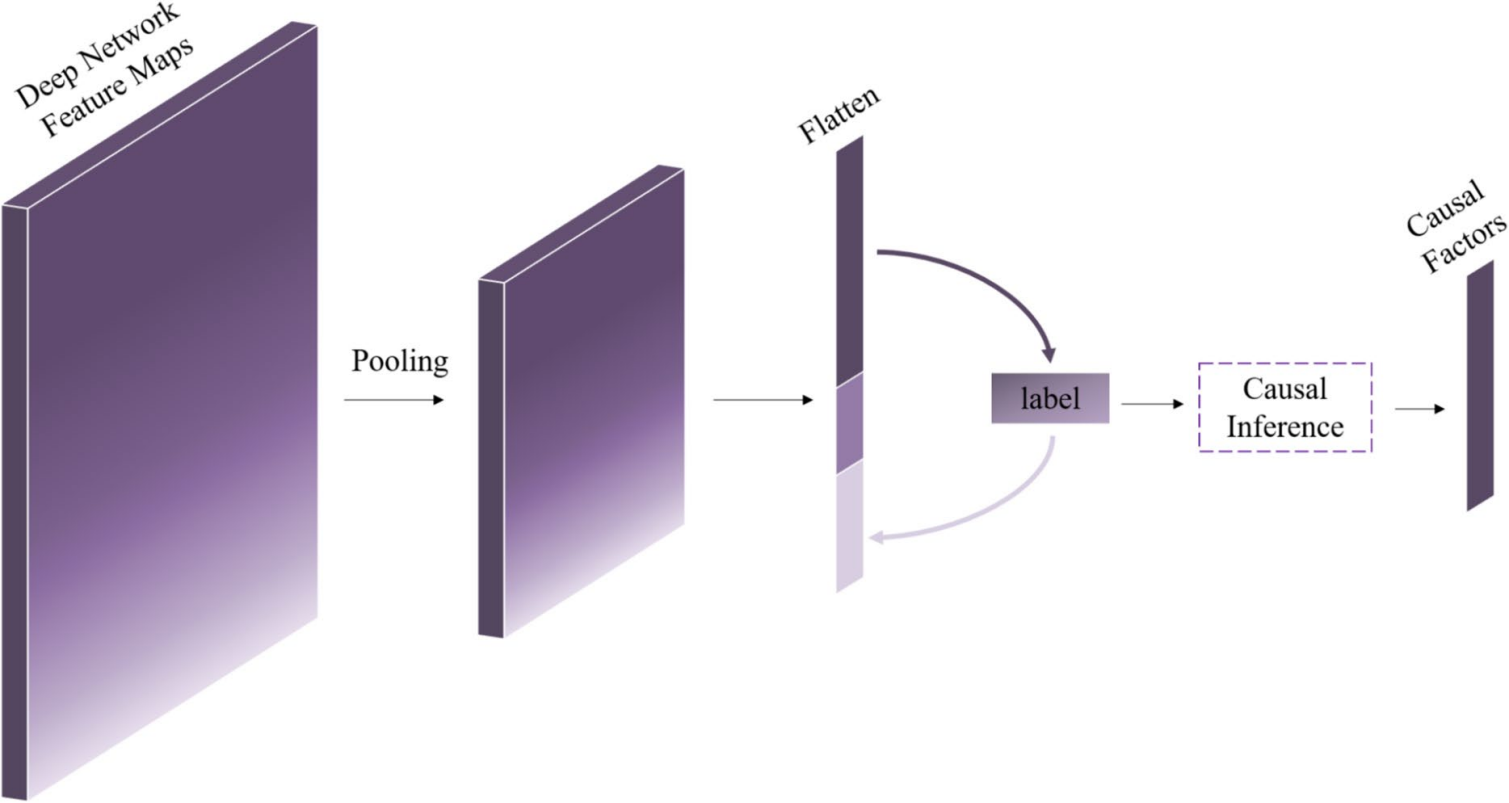
This diagram illustrates the process of identifying causal factors influencing the target variable from feature maps. The features depicted in the diagram can signify causes, effects of y, or maintain independence, as denoted by the arrows. Causal inference encompasses the examination of independence and conditional independence between y and attributes, thereby unveiling noteworthy causal factors

The conditional independence test acts as a versatile and robust causal inference method, utilizing the inherent conditional independence structures present in causal graphs. Within the scope of our current causal inference problem, we observe that a pair of agents can be considered causes of y only if their dependence is strengthened after conditioning on y. To operationalize this concept, we conduct independence and conditional independence tests for each pair of factors, with a focus on pairs where dependencies intensify after conditioning on y. This comparative analysis of test results facilitates the identification of such pairs. Importantly, these tests can be parallelized to enhance practical efficiency (Fig. 3).

Once the causal factors for the target y have been identified in each model, we progress to the concatenation layer. The causalities learned during flattening are integrated with the causal features and influence, achieved through fully connected layers, impacting the classification results.

Throughout the process of model learning, the system autonomously discerns which factors are crucial for accurate classification. This additional feature of the model represents a significant enhancement towards achieving improved classification accuracy, especially in the context of medical image datasets, where the logic behind image distribution among classes is contingent on the causal nature of the scenes depicted in the images.

### Classification with shape and texture features

In this study, we aimed to enhance and broaden the scope of our research by not only utilizing existing datasets but also by proactively collecting datasets directly from single cervix cells through the collaborative efforts of our research team. This exclusive dataset comprises invaluable information derived from authentic samples obtained from reputable sources. Upon data collection, we partitioned the cells into two distinct components: the nucleus and the cytoplasm, each exhibiting distinct characteristics. Through meticulous analysis of these segmented images, we identified and extracted meaningful and distinguishing features. Subsequently, we employed a combination of independence and conditional independence tests for every feature pair, coupled with advanced machine learning techniques such as K-Nearest Neighbors (KNN), Support Vector Machines (SVM), and Random Forest (RF), to accomplish precise cell classification. These models, driven by the extracted image-based features encompassing shape and texture attributes, demonstrated exceptional accuracy in recognizing and categorizing cells.

The extracted features in our analysis consist of two major components: shape features and texture features, the descriptions are briefly shown in Table 1.

**Table 1:** Features extracted during our analysis of images and corresponding descriptions

Feature Type	Feature Name	Description
Shape	Nucleus/Cytoplasm Area	This quantifies the number of pixels that make up the segmented image of the nucleus and cytoplasm
	Nucleus/Cytoplasm Ratio	This ratio provides insights into the relative sizes of the nucleus and cytoplasm, calculated as Nucleus Area divided by the sum of Nucleus Area and Cytoplasm Area
	Nucleus/Cytoplasmic Perimeter	It measures the length of the perimeter around the object, offering information about its shape and boundaries
	Nucleus/Cytoplasm Roundness	This metric gauge the roundness of the object by comparing its actual area to the area inside a circle defined by its longest diameter
	Nucleus/Cytoplasm Shortest Diameter	This represents the largest diameter a circle can have while being completely inscribed within the object
	Nucleus/Cytoplasm Longest Diameter	This signifies the shortest diameter a circle can have while fully circumscribing the object
	Nucleus/Cytoplasm Elongation	Elongation is determined by the ratio between the shortest and longest diameters of the object, providing insights into its elongated or compact nature
	Nucleus Position	This feature assesses how centrally the nucleus is positioned within the cytoplasm, offering information on the object's spatial distribution
Texture	Contrast	It quantifies the local variations in pixel intensity within the image. High contrast values indicate significant variations
	Correlation	This feature measures the likelihood of specific pixel pairs occurring, providing insights into the pixel relationship
	Energy	Energy is a statistical measure of the image's randomness or entropy, indicating the complexity of pixel patterns
	Homogeneity	Homogeneity assesses the similarity of pixel intensities across the image, offering insights into the uniformity of texture

## Experiments

### Experimental setup

In this experiment, we utilized the NVIDIA GeForce 3060 GPU for both training and testing our model. The experimental setup involves Python 3, which comes pre-configured with a suite of essential machine learning libraries including Tensorflow, Matplotlib, Keras, PyTorch, and OpenCV. These tools were integrated seamlessly within a Jupyter notebook environment.

### Dataset

#### SIPaKMeD

The SIPaKMeD dataset comprises 4049 annotated cell images. Expert cytopathologists have classified these cells into five different classes based on their cellular appearance and morphology. Specifically, normal cells are divided into two categories: superficial-intermediate and parabasal. Abnormal cells, which are not malignant, are further divided into two categories: koilocytes and dyskeratotic. Additionally, there is a category for benign cells, specifically metaplastic cells [58].

**Fig. 4:**
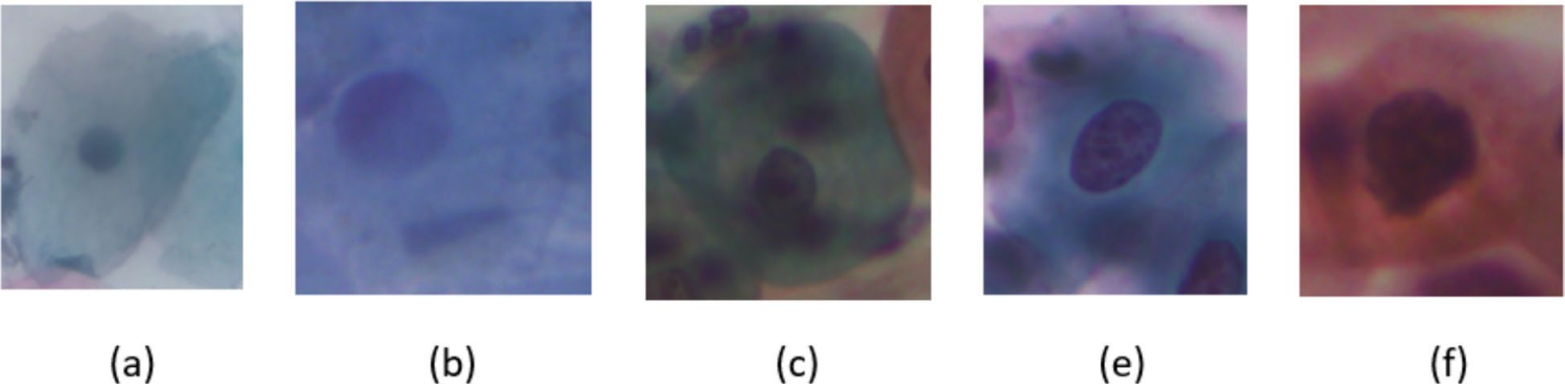
An example of SIPaKMeD database in five categories: **a** superficial-intermediate, **b** parabasal, **c** Metaplastic, **d** Koilocytes, **e** Dyskeratotic

The distribution of cells based on their classes is shown in Table 2. For visual examples of images from this dataset, refer to Fig. 4.

**Table 2:** Distribution of SIPaKMeD cells

	Category	Number of cells
Normal	Superficial-Intermediate	813
	Parabasal	787
Benign	Metaplastic	793
Abnormal	Koilocytes	825
	Dyskeratotic	813
Total		4049

#### ShUCSEIT

The data were collected from vaginal smears using a light microscope (Olympus DP-72) with a fixed magnification at × 40. Each image captured encompasses multiple cells, some of which may overlap. The cell types diagnosis were confirmed by expert pathologist. To isolate individual cells, the images with non-overlapping cells were segmented and saved as individual entities. Based on their appearance and cell morphology, the microscopic images were categorized into five distinct groups: Superficial squamous epithelial, Intermediate squamous epithelial, Parabasal squamous epithelial, low-grade squamous intraepithelial lesion (LSIL), and high-grade squamous intraepithelial lesion (HSIL). Among these categories, superficial, intermediate, and parabasal cells are regarded as normal cells, while LSIL and HSIL cells are classified as abnormal cells.

**Fig. 5:**
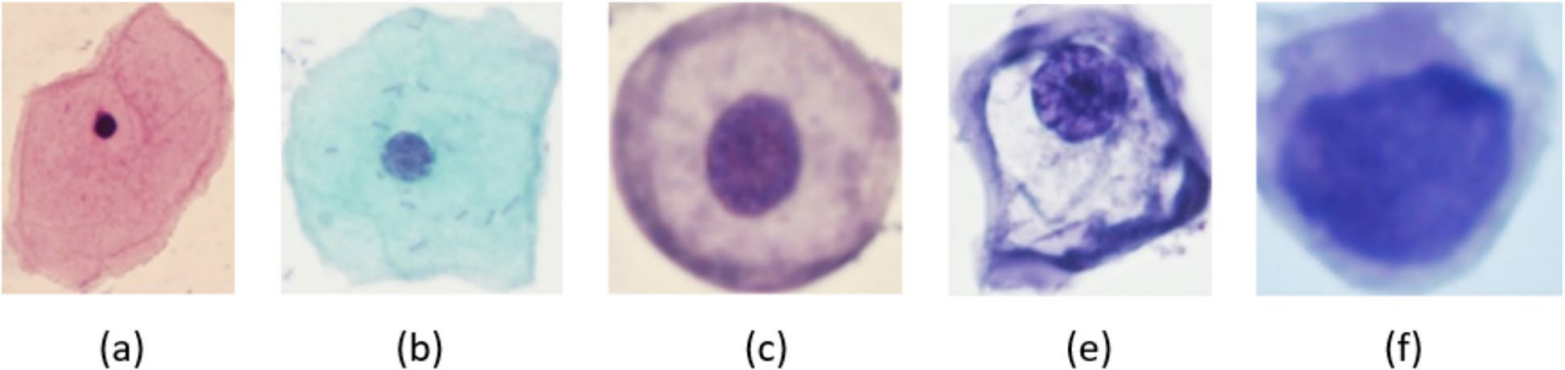
An example of ShUCSEIT database in five categories: **a** Superficial, **b** Intermediate, **c** Parabasal, **d** LSIL, **e** HSIL

The distribution of cells based on their classes is shown in Table 3. For visual examples of images from this dataset, refer to Fig. 5.

**Table 3:** Distribution of ShUCSEIT cells

	Category	Number of cells
Normal	Superficial squamous epithelial	507
	Intermediate squamous epithelial	423
	Parabasal squamous epithelial	444
Abnormal	Low-grade squamous intraepithelial lesion	406
	High-grade squamous intraepithelial lesion	420
Total		2201

#### Herlev

**Fig. 6:**
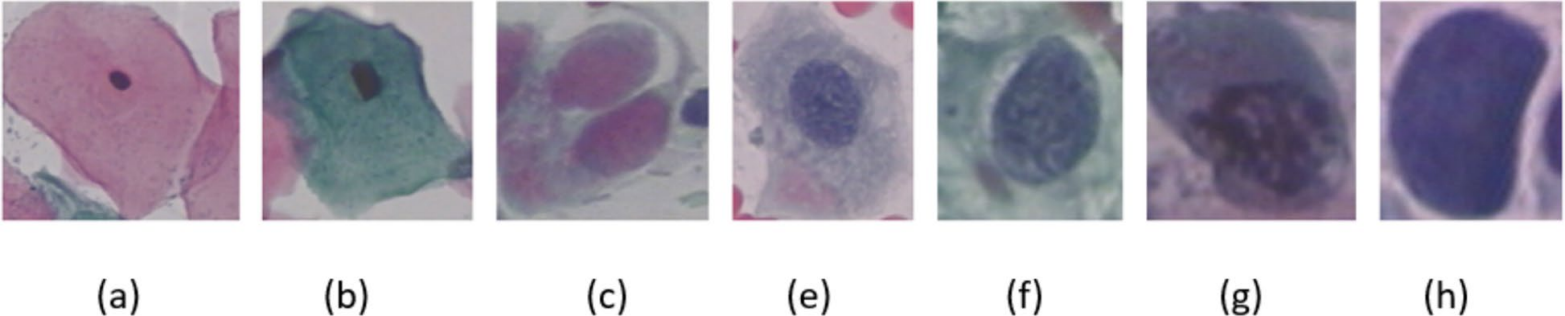
An example of Herlev database in seven categories: **a** Superficial squamous epithelial, **b** Intermediate squamous epithelial, **c** Columnar epithelial, **d** Mild squamous non-keratinizing dysplasia, **e** Moderate squamous non-keratinizing dysplasia, **g** Severe squamous non-keratinizing dysplasia, **h** Squamous cell carcinoma in situ intermediate

The Herlev Pap-smear dataset represents the latest iteration of two versions developed by Herlev University Hospital. Skilled staff at the hospital meticulously prepared and analyzed the images, employing CHAMP (Dimac), a commercial software package, for image segmentation. The cell selection process prioritized the inclusion of crucial classes rather than adhering to a natural distribution [59]. The distribution of cells based on their classes is shown in Table 4. For visual examples of images from this dataset, refer to Fig. 6.

**Table 4:** Distribution of Herlev cells

	Category	Number of cells
Normal	Superficial squamous epithelial	74
	Intermediate squamous epithelial	70
	Columnar epithelial	98
Abnormal	Mild squamous non-keratinizing dysplasia	182
	Moderate squamous non-keratinizing dysplasia	146
	Severe squamous non-keratinizing dysplasia	197
	Squamous cell carcinoma in situ intermediate	150
Total		917

### Evaluation method

Assessing the performance of a machine learning model is a crucial task in its development. Precision, recall, F1-score, and accuracy are widely recognized as standard measures for evaluating classification performance [60].

The precision metric measures the number of correctly identified samples among all recognized representations. On the other hand, recall defines the ability of a classification model to recognize all the relevant samples. The F1-score combines both precision and recall by using the harmonic mean. Accuracy, on the other hand, represents the proportion of correctly predicted samples out of the total number of samples. The ROC (Receiver Operating Characteristic) is a probability curve that graphically illustrates the True Positive Rate (TPR) in relation to the False Positive Rate (FPR). Meanwhile, the AUC (Area Under the Curve) is a single scalar value that quantifies the classifier's performance by summarizing the information contained within the ROC curve. The mathematical expressions for these evaluation metrics are provided in Table 5.

**Table 5:** Evaluation metrics

Metric	Formula
Precision	$\frac{TP}{TP + FP}$
Recall	$\frac{TP}{TP + FN}$
F1-score	$2\times \frac{Precision \times Recall }{Precision + Recall}$
Accuracy	$\frac{TP+ TN}{TP+TN+FP+FN}$
AUC	$\frac{TruePositiveRate- FalsePositiveRate+1}{2}$

True positive (TP) denotes the number of accurately labeled positive samples, while true negative (TN) represents the number of correctly classified negative samples. False positive (FP) refers to the number of negative samples classified as positive, and false negative (FN) represents the number of positive instances predicted as negative [61].

### Data setting

The cervical cell images used in evaluating the efficacy of our proposed methodology exhibit diverse dimensions. To ensure uniformity in image size for subsequent analysis and processing, each image has been consistently resized to (224 × 224) pixels. This crucial resizing step guarantees consistency and facilitates rigorous analysis and processing of the images. To enhance the model's performance, data augmentation techniques were exclusively applied to the training sets. Techniques include geometric transformations, color space changes, kernel filters, random erasing, and image mixing. TensorFlow (TF) and Keras provide built-in methods for augmentation. As a result, the training datasets for SIPaKMeD and ShUCSEIT were augmented by a factor of 6, while the training dataset for Herlev was augmented by a factor of 14. For this study, we adopted a standard data split strategy for each dataset, allocating 60% of the data in each class for training, 20% for validation, and 20% for testing. The classification tasks involved both 5-class datasets (SIPaKMeD and ShUCSEIT) and a 7-class dataset (Herlev). More detailed information about the resulting training, validation, and test datasets can be found in Table 6.

**Table 6:** Experimental distribution of datasets

	Number of Images		
	ShUCSEIT	SIPaKMeD	Herlev
Train	9240	16,982	8190
Validation	440	811	185
Test	440	812	186

To establish a cohesive perspective on the data classes, we can assert that the Superficial and Intermediate classes are analogous to the Superficial and Intermediate categories within other datasets. Specifically, the Parabasal class aligns with the Metaplastic and Parabasal classes found in the SIPaKMeD dataset. Meanwhile, the LSIL class corresponds to the Mild category in the Herlev dataset and the Koilocytes category in the SIPaKMeD dataset. Lastly, the HSIL class equates to the Dyskeratotic class in the SIPaKMeD dataset, as well as the Moderate, Severe, and carcinoma in situ categories in the Herlev dataset.

### Data analysis

In this study, we leveraged the extensive ShUCSEIT dataset to conduct a comprehensive analysis of cellular images. Our primary objective was to accurately segment nucleus, cytoplasm, and cell boundaries, employing advanced computer vision techniques (Table 7). This segmentation process laid the foundation for extracting shape-based features (Table 1). These descriptors encapsulated the geometric properties of the cellular structures, providing a robust basis for subsequent classification efforts.

**Table 7:**
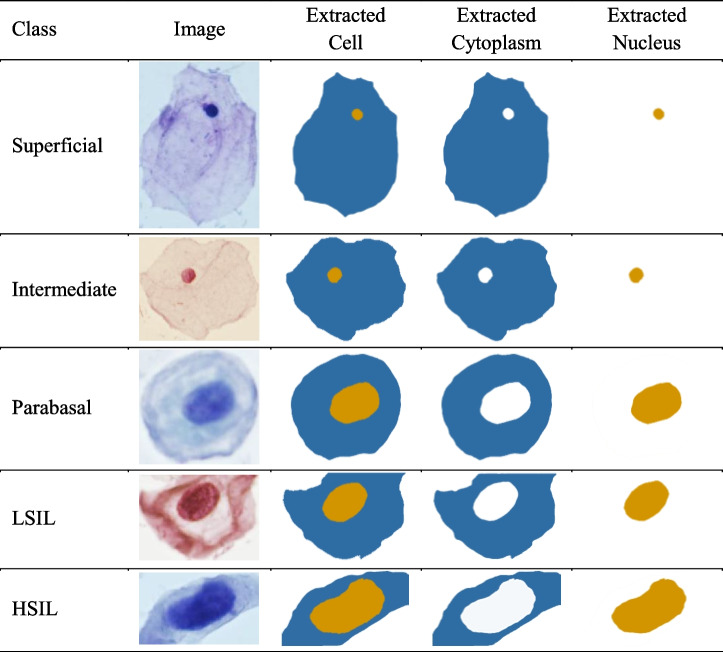
Examples of our segmentation results for cell, nucleus, and cytoplasm

With the extracted shape-based features in hand, we proceeded to employ machine learning techniques for image classification. The results, as depicted in Tables 8 and 9, showcased the efficacy of our approach. The classification metrics, including accuracy, precision, recall, AUC, and F1-score, provided a thorough assessment of the model's performance. This study demonstrates the promising potential of shape-based features in accurate cellular image classification, paving the way for further advancements in this critical domain of biomedical research.

**Table 8:** The performance analysis of the presented methods on the ShUCSEIT dataset

Models	Avg Precision	Avg Recall	Avg F1-score	Avg Accuracy	AUC
KNN	0.764	0.706	0.675	71.59%	0.822
SVM	**0.847**	**0.846**	**0.843**	**84.09%**	**0.901**
RF	0.807	0.808	0.807	80.68%	0.879

**Table 9:** The performance analysis of the presented methods using independence and conditional independence tests on the ShUCSEIT dataset

Models	Avg Precision	Avg Recall	Avg F1-score	Avg Accuracy	AUC
KNN	0.814	0.778	0.769	78.41%	0.865
SVM	**0.897**	**0.896**	**0.894**	**89.09%**	**0.932**
RF	0.851	0.852	0.851	85.00%	0.906

As evident from the observed results, the incorporation of causal discovery methods led to significant enhancements in our findings. This improvement in accuracy and precision underscores the importance of leveraging advanced methodologies in cellular image analysis. The combination of these techniques not only refines our understanding of cellular structures but also holds immense potential for broader applications in biomedical research and clinical practice.

### Experimental results

In this study, we conducted a rigorous assessment of the CausalCervixNet framework, benchmarking its performance against well-established deep learning architectures (VGG16, VGG19, ResNet50, and XceptionNet) for automated cervical cell classification. The evaluation was performed on unseen test datasets encompassing both 5-class (SIPaKMeD and ShUCSEIT) and 7-class (Herlev) classification tasks. The results are systematically detailed in Tables 10 and 11.

**Table 10:** The performance analysis of the base models

Dataset	Models	Avg. Precision	Avg. Recall	Avg. F1-score	Avg. Accuracy	AUC	Time (sec)
**SIPaKMeD**	VGG16	**0.978**	**0.978**	**0.978**	**97.78%**	**0.986**	5200
	VGG19	0.963	0.962	0.962	96.18%	0.976	5500
	ResNet-50	0.949	0.948	0.948	94.82%	0.968	6000
	XceptionNet	0.760	0.656	0.649	65.64%	0.785	6400
**ShUCSEIT**	VGG16	0.939	0.937	0.938	93.64%	0.960	3200
	VGG19	0.929	0.929	0.929	92.73%	0.955	3400
	ResNet-50	**0.965**	**0.964**	**0.964**	**96.36%**	**0.977**	3800
	XceptionNet	0.857	0.857	0.857	85.45%	0.909	4200
**Herlev**	VGG16	0.616	0.628	0.605	60.21%	0.768	5200
	VGG19	0.659	0.635	0.643	59.14%	0.762	5400
	ResNet-50	**0.825**	**0.826**	**0.824**	**81.18%**	**0.890**	5800
	XceptionNet	0.434	0.431	0.387	40.32%	0.652	6200

**Table 11:** The performance analysis of the proposed CausalCervixNet method

Dataset	Models	Avg. Precision	Avg. Recall	Avg. F1-score	Avg. Accuracy	AUC	Time (sec)
**SIPaKMeD**	CICNN-VGG16	0.988	0.987	0.988	98.76%	0.992	10,000
	CICNN-VGG19	0.986	0.985	0.985	98.52%	0.991	10,500
	CICNN-ResNet-50	**0.991**	**0.991**	**0.991**	**99.14%**	**0.995**	11,200
	CICNN-XceptionNet	0.881	0.861	0.862	86.21%	0.914	11,800
**ShUCSEIT**	CICNN-VGG16	0.971	0.971	0.971	97.05%	0.982	6300
	CICNN-VGG19	0.969	0.970	0.970	96.82%	0.980	6700
	CICNN-ResNet-50	**0.991**	**0.991**	**0.991**	**99.09%**	**0.994**	7400
	CICNN-XceptionNet	0.933	0.933	0.932	93.18%	0.957	8000
**Herlev**	CICNN-VGG16	0.898	0.860	0.871	86.02%	0.918	3600
	CICNN-VGG19	0.876	0.853	0.859	84.95%	0.912	3900
	CICNN-ResNet-50	**0.979**	**0.978**	**0.978**	**97.31%**	**0.984**	4500
	CICNN-XceptionNet	0.837	0.786	0.793	79.57%	0.881	5000

Table 10 presents the comparative performance of the deep learning models. XceptionNet demonstrated the lowest classification accuracy across all datasets, while VGG16 yielded the best performance for the SIPaKMeD dataset. In contrast, ResNet50 exhibited superior generalization capabilities, outperforming the other models on the ShUCSEIT and Herlev datasets. This result underscores the adaptability of ResNet50 in handling diverse cytological image variations.

Table 11 expands on these findings by illustrating the performance of CausalCervixNet, which integrates each of the four network architectures (VGG16, VGG19, ResNet50, and XceptionNet) within a causality-aware classification pipeline. ResNet50 consistently surpassed all competing models, achieving the highest classification metrics across datasets. Specifically, ResNet50 attained an accuracy of 99.14%, a precision of 0.991, a recall of 0.991, and an F1-score of 0.991 on the SIPaKMeD dataset, emphasizing its robustness in cervical cytopathology classification.

**Fig. 7:**
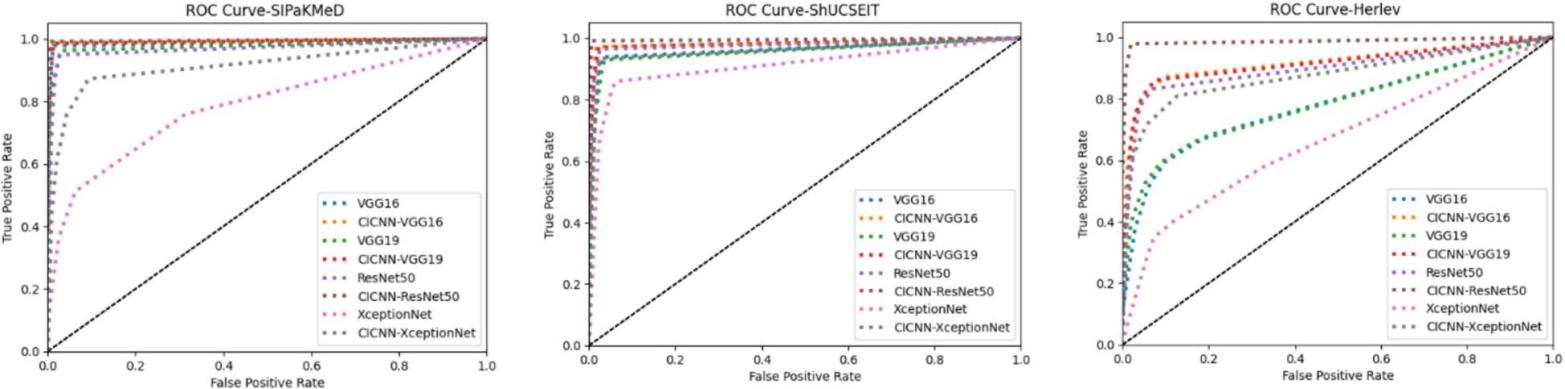
ROC curves depict the performance of various classification models in distinguishing cervical cell images across diverse datasets

Crucially, CausalCervixNet demonstrated a significant performance advantage over traditional deep learning models that lack causal inference capabilities, reinforcing its effectiveness in enhancing classification accuracy and interpretability. Figure 7 visually represents the ROC curves, illustrating the superior discriminative power of CausalCervixNet across all datasets.

These findings underscore the transformative potential of integrating causal inference with deep learning in medical image classification. Our results highlight the necessity of strategic model selection and the incorporation of causal reasoning methodologies to advance the reliability and transparency of AI-driven diagnostic systems. The superior performance of CausalCervixNet validates its applicability as an advanced, interpretable, and highly effective framework for cervical cytology classification.

**Fig. 8:**
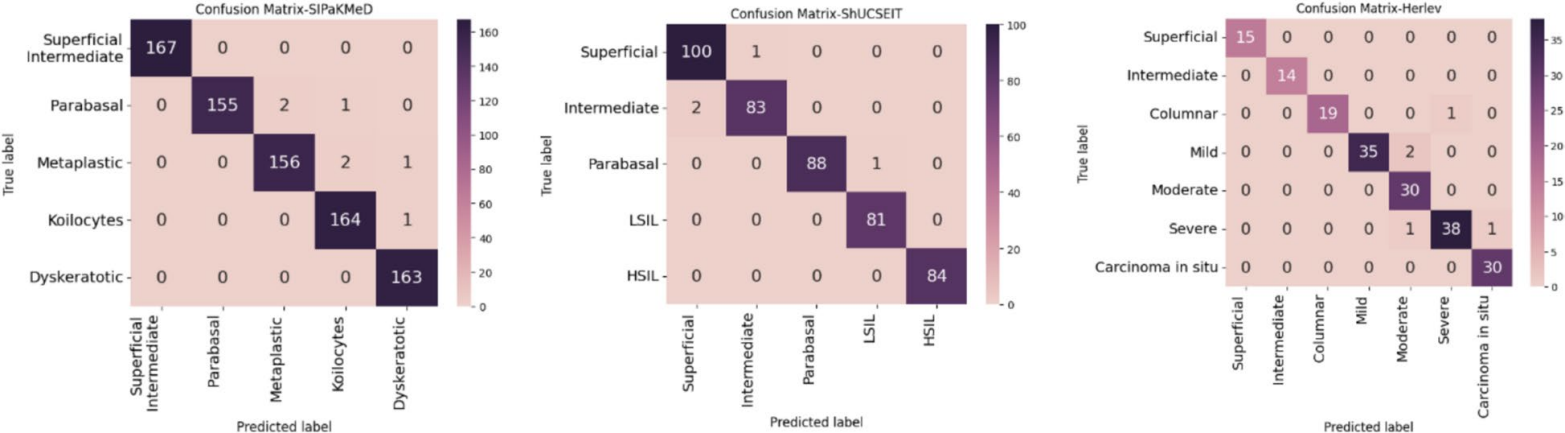
The confusion matrix depicts the results of the CausalCervixNet method applied to the classification problem using the SIPaKMeD, ShUCSEIT and Herlev datasets

Finally, Fig. 8 presents the confusion matrices generated by CausalCervixNet, further illustrating its precision and dependability in distinguishing between different cervical cell types. These results collectively reinforce the robustness of causality-driven deep learning models in medical image analysis, paving the way for more trustworthy AI applications in clinical diagnostics.

## Discussion

In this study, we introduced and examined the CausalCervixNet method for cervical cell classification on the SIPAKMED, ShUCSEIT, and Herlev datasets. Additionally, we proposed a causal inference scheme to identify the causal factors influencing the target. Leveraging these causal factors, our method demonstrated superior performance (Table 11). The results of our approach highlight the potential of causal inference in enhancing the accuracy and effectiveness of cervical cell classification.

Based on Fig. 8 and the confusion matrix results from all three datasets, it is evident that there were no instances where cancerous cells were misdiagnosed as non-cancerous. For example, in ShUCSEIT dataset, intermediate cell misdiagnosed as a superficial cell 2 times and superficial cell misdiagnosed as intermediate cell one time. Also, in one case parabasal cells (normal cell) diagnosed as LSIL (abnormal cell). The same results are seen in 2 other datasets. This finding holds significant importance in medical cases. For instance, if a patient with cancerous cells were wrongly identified as non-cancerous, they might stop their treatment, leading to disease progression with potentially dangerous consequences. On the other hand, if the opposite scenario occurs, they can undergo appropriate follow-up and testing, resulting in fewer negative consequences.

Table 12 provides examples of misclassified cervical cells on the SIPAKMED, ShUCSEIT and Herlev dataset for classification. It was found that three misclassifications occurred in the ShUCSEIT dataset images, specifically within the Intermediate and Superficial classes. These two classes are normal cells and exhibit a high degree of similarity, which aligns with the pathologists' opinion, stating the likelihood of errors between these classes.

**Table 12:**
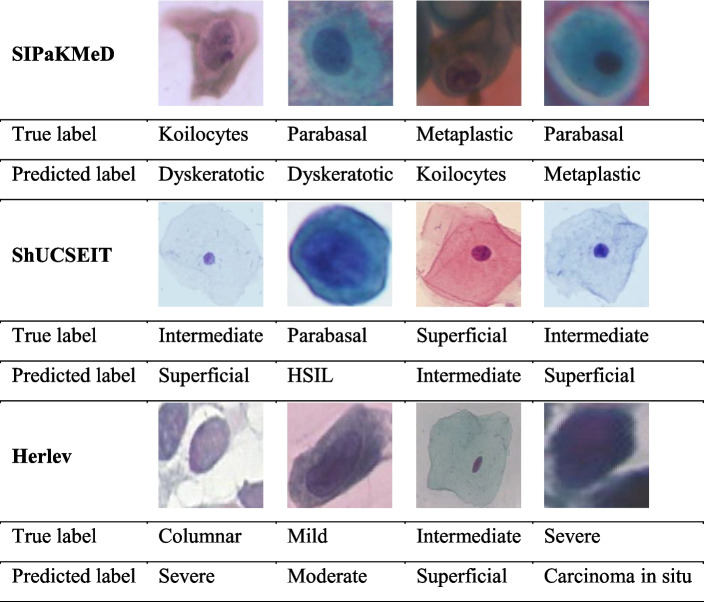
Illustrations of cervical cells misclassified by CausalCervixNet method

In summary, CausalCervixNet combines preprocessing, feature extraction, causality map, causal inference, and classification stages to identify causal factors and achieve accurate classification, making it a valuable tool for causal analysis and predictive modeling.

XceptionNet's accuracy on the Herlev dataset was initially low at 40%, but it increased significantly to 80% with the integration of CICNN. This improvement is attributed to CICNN's ability to utilize causality-based insights, which enhance the identification of relevant features while reducing the impact of noise and spurious correlations. By incorporating a causality map and leveraging causal inference, the model effectively prioritizes meaningful feature relationships, particularly in datasets with complex class structures and imbalances.

In contrast, while HDFF achieves comparable accuracy on the SIPaKMeD dataset, it lacks CICNN's capability to refine feature importance through causal discovery. This limitation is particularly evident in datasets like Herlev, where CICNN demonstrated superior robustness in addressing inter-class similarities and variability. These results highlight the critical role of causality-driven methods in enhancing generalization and interpretability, as shown in Tables 10 and 11.

We evaluated the diversity of the SIPaKMeD, Herlev, and ShUCSEIT datasets, with the latter specifically designed to enhance representation through varied staining techniques and cellular characteristics. Despite applying data augmentation and causal inference methods to mitigate biases, demographic factors such as ethnicity, age, and geography may impact model generalizability. We acknowledge this limitation and emphasize the need for broader demographic representation in future research to improve model robustness and clinical applicability.

In Table 13, we provide a thorough comparative analysis of existing methodologies and our novel approach across two distinct datasets. Our method stands out as the leading performer on the Herlev dataset, demonstrating results that significantly surpass those of competing methods. The substantial performance enhancement, as indicated by the notable margin between our outcome and those of alternative approaches, underscores the robustness and efficacy of our proposed methodology. Regarding the SIPaKMeD dataset, the HDFF method secures the top position, albeit with a marginal 0.01% difference from our result. While HDFF exhibits commendable performance, it is essential to highlight that the slight variance in outcomes falls within an acceptable range. What distinguishes our method, however, is the incorporation of causality, leading to more favorable results, as illustrated in Fig. 8. The emphasis on logical consistency is crucial in medical applications, and our findings underscore the significance of not solely relying on quantitative metrics for network evaluation.

**Table 13:** Comparison of the proposed method and existing methods on SIPaKMeD and Herlev datasets

		**SIPaKMeD**	**Herlev**
	**Models**	**Accuracy**	**Accuracy**
	HDFF [16]	**99.15%**	90.32%
	CerviFormer [21]	93.70%	94.57%
	DeepCell [18]	95.63%	92.72%
Our method	CICNN-VGG16	98.76%	86.02%
	CICNN-VGG19	98.52%	84.95%
	CICNN-ResNet-50	99.14%	**97.31%**
	CICNN-XceptionNet	86.21%	79.57%

## Limitations

While the integration of causal inference in cervical cancer classification presents significant advantages, there are inherent challenges and limitations that must be acknowledged.

- Constraints of Causal Inference in High-Dimensional Data
  Causal inference methods often struggle when applied to high-dimensional datasets, such as medical imaging data, due to the following reasons:
  - Computational Complexity: Estimating causal relationships among a large number of features requires significant computational power. Many causal discovery algorithms rely on independence tests, graph-based models, or structural equation modeling, all of which become increasingly expensive as the number of variables grows.
  - Spurious Correlations: High-dimensional datasets tend to exhibit a large number of correlated features, which can introduce spurious causal links. Distinguishing between true causal relationships and coincidental associations remains a significant challenge.
  - Data Sparsity and Limited Samples: Despite access to large image datasets, the number of unique, well-labeled samples remains limited compared to the feature space. This can lead to overfitting in causal models and difficulties in generalizing to unseen cases.
  - Latent Confounders: Unobserved variables that influence both the cause and effect can distort causal inference. In medical imaging, variations in staining, imaging conditions, or patient demographics may introduce hidden biases.
- Challenges in Implementation
  Implementing causality-driven deep learning models, such as CausalCervixNet, introduces several practical difficulties:
  - Integration with Deep Learning Architectures: Combining causal inference with convolutional neural networks (CNNs) requires careful alignment of feature representations with causal estimation methods. Conventional deep learning models are optimized for feature extraction but are not inherently designed for causal reasoning.
  - Trade-off Between Interpretability and Performance: While causal inference enhances model interpretability, integrating causality-based approaches can lead to a slight increase in computational cost, potentially impacting real-time medical diagnosis applications.
  - Validation and Benchmarking: Unlike traditional classification models that rely solely on accuracy metrics, evaluating the success of a causality-based model requires additional validation, such as assessing the correctness of identified causal relationships. Standardized benchmarks for causality-enhanced medical AI models are still underdeveloped.

## Conclusions

This study introduces CausalCervixNet, a novel deep learning and causality-based method for classifying cervical cells. By estimating pairwise causal relationships between features and identifying the causal factors of the target variable, the proposed framework integrates a causal inference scheme employing conditional probabilities, independence tests, and causal discovery algorithms. The results demonstrate that CICNN-ResNet-50 significantly outperforms other approaches, achieving higher classification accuracies and setting new benchmarks in cervical cell classification. Specifically, the model achieved state-of-the-art accuracies of 99.14% for the 5-class classification problem on the SIPaKMeD dataset and 99.09% on the ShUCSEIT dataset, while attaining an accuracy of 97.31% for the 7-class classification problem on the Herlev dataset.

The success of this study lies in its innovative integration of causal reasoning into deep learning, which goes beyond traditional methods that rely solely on statistical dependence. By identifying and leveraging causal relationships between features, CausalCervixNet enhances both interpretability and robustness, addressing key challenges in real-world medical imaging. Additionally, the framework's ability to generalize across diverse datasets, its computational efficiency through parallelized causality testing, and its capacity to handle imbalanced datasets through advanced feature fusion techniques further highlight its superiority over similar studies.

This work not only achieves exceptional classification performance but also provides a transparent and interpretable solution for cervical cancer diagnostics, which is crucial for clinical applications. By shedding light on the potential benefits of causality-based approaches, this research paves the way for future studies to explore the intersection of deep learning and causal inference in medical image analysis. The findings underscore the promise of using these techniques to enhance accuracy, generalizability, and explainability in healthcare, particularly in the context of cervical cell classification.

## Data Availability

The datasets generated and/or analyzed during the current study are available from the corresponding author upon reasonable request. If a citation is provided, we will share the data accordingly.

## Abbreviations

AI: Artificial Intelligence
ML: Machine Learning
DL: Deep Learning
CICNN: Causal Insight Convolutional Neural Network
CNN: Convolutional Neural Network
DFF: Deep Feature Fusion
HSIC: Hilbert-Schmidt Independence Criterion
KCIT: Kernel Conditional Independence Test
KCI: Kernel Conditional Independence
HSIL: High-Grade Squamous Intraepithelial Lesion
LSIL: Low-Grade Squamous Intraepithelial Lesion
SVM: Support Vector Machine
RF: Random Forest
KNN: K-Nearest Neighbors
TL: Transfer Learning
TN: True Negative
TP: True Positive
FN: False Negative
FP: False Positive
ROC: Receiver Operating Characteristic
AUC: Area Under the Curve
